# Calling and Phasing of Single-Nucleotide and Structural Variants of the *LDLR* Gene Using Oxford Nanopore MinION

**DOI:** 10.3390/ijms24054471

**Published:** 2023-02-24

**Authors:** Maria S. Nazarenko, Aleksei A. Sleptcov, Aleksei A. Zarubin, Ramil R. Salakhov, Alexander I. Shevchenko, Narek A. Tmoyan, Eugeny A. Elisaphenko, Ekaterina S. Zubkova, Nina V. Zheltysheva, Marat V. Ezhov, Valery V. Kukharchuk, Yelena V. Parfyonova, Suren M. Zakian, Irina S. Zakharova

**Affiliations:** 1 Research Institute of Medical Genetics, Tomsk National Research Medical Center, Russian Academy of Sciences, Tomsk 634050, Russia; 2 Institute of Cytology and Genetics, Siberian Branch of Russian Academy of Sciences, Novosibirsk 630090, Russia; 3 Federal State Budgetary Institution National Medical Research Center of Cardiology Named after Academician E.I. Chazov, Ministry of Health of Russian Federation, Moscow 121552, Russia

**Keywords:** LDLR, Oxford Nanopore, familial hypercholesterolemia, structural variant, haplotype

## Abstract

The *LDLR* locus has clinical significance for lipid metabolism, Mendelian familial hypercholesterolemia (FH), and common lipid metabolism-related diseases (coronary artery disease and Alzheimer’s disease), but its intronic and structural variants are underinvestigated. The aim of this study was to design and validate a method for nearly complete sequencing of the *LDLR* gene using long-read Oxford Nanopore sequencing technology (ONT). Five PCR amplicons from *LDLR* of three patients with compound heterozygous FH were analyzed. We used standard workflows of EPI2ME Labs for variant calling. All rare missense and small deletion variants detected previously by massively parallel sequencing and Sanger sequencing were identified using ONT. One patient had a 6976 bp deletion (exons 15 and 16) that was detected by ONT with precisely located breakpoints between *AluY* and *AluSx1*. *Trans*-heterozygous associations between mutation c.530C>T and c.1054T>C, c.2141-966_2390-330del, and c.1327T>C, and between mutations c.1246C>T and c.940+3_940+6del of *LDLR*, were confirmed. We demonstrated the ability of ONT to phase variants, thereby enabling haplotype assignment for *LDLR* with personalized resolution. The ONT-based method was able to detect exonic variants with the additional benefit of intronic analysis in one run. This method can serve as an efficient and cost-effective tool for diagnosing FH and conducting research on extended *LDLR* haplotype reconstruction.

## 1. Introduction

The *LDLR* gene encodes the low-density lipoprotein (LDL) receptor protein, which is responsible for receptor-mediated endocytosis of LDL particles, mainly by hepatocytes, and thus maintains the plasma level of LDL. To date, more than 18,000 variants, including 3000 rare variants, have been identified in the *LDLR* gene [1,2,3].

Common polymorphisms of this gene are associated with abnormal serum lipid levels, coronary artery disease (CAD), angina pectoris, myocardial infarction, abdominal aortic aneurysm, and Alzheimer’s disease, according to genome-wide association studies (GWASs) [4]. Rare pathogenic variants in the *LDLR* gene cause a type of high blood cholesterol called familial hypercholesterolemia (FH) and are responsible for approximately 84% of FH cases [5]. These mutations have been subdivided into five classes based on biochemical and functional studies on *LDLR* variants [6]. Most patients with FH have heterozygous loss-of-function mutations in *LDLR*. In rare cases, homozygous FH results from homozygous or, more often, from compound heterozygous mutations in the *LDLR* gene [7,8].

The vast majority of FH patients carry a missense mutation which arises from a single-nucleotide variant (SNV) in the coding region of the *LDLR* gene and affects protein structure and function [6]. Intronic variants of this gene may also impact the disease phenotype [9,10]. According to some studies, structural variants (SVs) account for approximately 10% of mutations in the *LDLR* gene [11,12]. This finding emphasizes the need to broaden the scope of this research from coding regions of the *LDLR* gene to complete *LDLR* gene sequencing that identifies all types of genetic variants, such as SNVs and SVs and including haplotype reconstruction, in one run, especially in patients with a yet unknown genetic cause of FH.

Long-read DNA sequencing methods, specifically Oxford Nanopore technology (ONT), have advanced medical genetics by enabling the rapid and low-cost assessment of targeted genes, or even of the clinical exome, by detecting SVs and accurately determining haplotypes [13,14,15,16]. Recently, Soufi M. et al. presented a nanopore-sequencing-based workflow for rapid genetic testing of FH in a clinical service laboratory [15]. They amplified the *LDLR* gene in five fragments, covering the promoter region and coding sequences of all 18 exons. Therefore, this workflow may miss patients with deep intronic variants. There is also a problem with the phasing of genetic variants and direct haplotype analysis in the case of compound heterozygosity. Such information is important not only for index patients to confirm the FH diagnosis but also for potential diagnostic tools, preventative lifestyle interventions, and therapeutic management of family members to reduce their risk of CAD.

For these reasons, we aimed to evaluate nanopore sequencing for calling and phasing SNVs and SVs of the *LDLR* gene. As a result, a workflow of long-range amplification of the *LDLR* gene comprising all types of genetic variants from exon 2 to exon 18 with introns was developed and validated on monomolecular sequencing technology. We applied the method to three patients with compound heterozygous mutations in the *LDLR* gene. We demonstrated that complete resolution of all variant types in *LDLR* by targeted ONT sequencing is possible. The advantage of long-read sequencing is direct and precise identification of a haplotype of the *LDLR* gene.

## 2. Results

### 2.1. Long-Range PCR Primers for Amplifying the LDLR Gene from Exon 2 to Exon 18 with Introns

**Figure 1:**
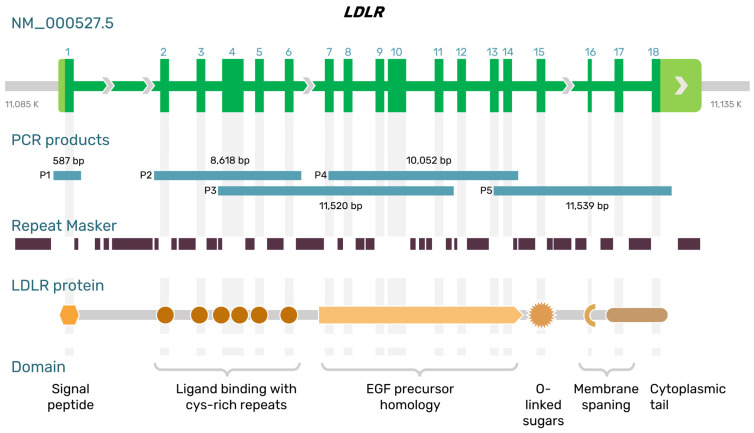
The structure of the *LDLR* gene and protein. Long-range PCR amplicons (P1–P5) in this study are illustrated by blue lines and size (bp).

The *LDLR* gene is located on the short arm of chromosome 19 (19p13.2) [17]. This gene spans ~45 kb of genomic DNA and contains 18 exons. The transcript (GenBank accession No. NM_000527.5) is 5.173 kb long and encodes a peptide consisting of 860 amino acids, including a 21-residue signal peptide [18]. Exon 1 of *LDLR* comprises a signal sequence that localizes the receptor to the endoplasmic reticulum for transport to the cell surface. The other exons encode five domains of LDLR: the ligand-binding domain (exons 2–6), epidermal growth factor (EGF) precursor homology domain (exons 7–14), a domain with O-linked carbohydrates (exon 15), a membrane-spanning domain, and a cytoplasmatic part of the receptor (exons 16–18; Figure 1).

Primer pair 1 is designed to amplify the promoter and exon 1 of the *LDLR* gene (Figure 1). The first amplicon’s length is 587 bp. Primer pairs 2, 3, 4, and 5 were used to detect exons 2–6, 4–11, 7–14, and 13–18, respectively. There were considerable overlaps between the amplicons, and the total amplicon size was 42,316 bp.

We conducted long-range PCR to amplify four fragments (P2, P3, P4, and P5) of the *LDLR* sequence from three unrelated probands that each carry two pathogenic variants in this gene. The large size (>10 kb) and the complexity of intron 1 prevented its efficient long-range amplification. Therefore, the promoter region, including exon 1, was amplified by classic PCR (587 bp) and analyzed by Sanger sequencing.

### 2.2. Nanopore Sequencing of SNVs and Small and Large Deletions in the LDLR Gene 

Sequencing of the pooled PCR products of each of the three DNA samples on one MinION flow cell yielded an average of 21,450 reads per sample. The mean read length was 7372 bp and the GC content was 52%. Mapping the reads to the human reference genome showed an average coverage of 5297× per sample around the *LDLR* region. Mean mapping quality of three DNA samples was 59.28.

Using the long-range PCR of four fragments of the *LDLR* gene and nanopore sequencing, we correctly identified all six pathogenic variants and their correct zygosity in the three DNA samples in which variants had previously been detected by massively parallel sequencing (MPS) or Sanger sequencing.

#### 2.2.1. SNVs and a Small Deletion

**Figure 2:**
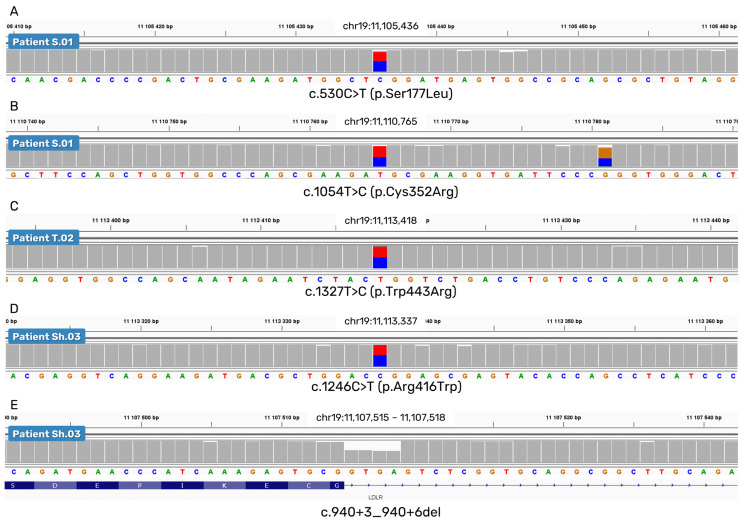
Missense and short deletion variants of the *LDLR* gene were detected by nanopore sequencing. A trace from the IGV software is shown for each sample carrying a mutation. (**A**,**B**) c.530C>T and c.1054T>C of patient S.01; (**C**) c.1327T>C of patient T.02; (**D**,**E**) c.1246C>T and c.940+3_940+6del of patient Sh.03.

In all three DNA samples analyzed, we identified four heterozygous pathogenic exonic SNVs (c.530C>T, c.1054T>C, c.1246C>T, and c.1327T>C) and one heterozygous likely pathogenic short deletion in intron 6: c.940+3_940+6del (Figure 2).

#### 2.2.2. A Deletion of Exons 15 and 16

*LDLR* is especially susceptible to SVs with breakpoints that are typically located within introns owing to the high density of Alu repeats [11,12,19,20].

**Figure 3:**
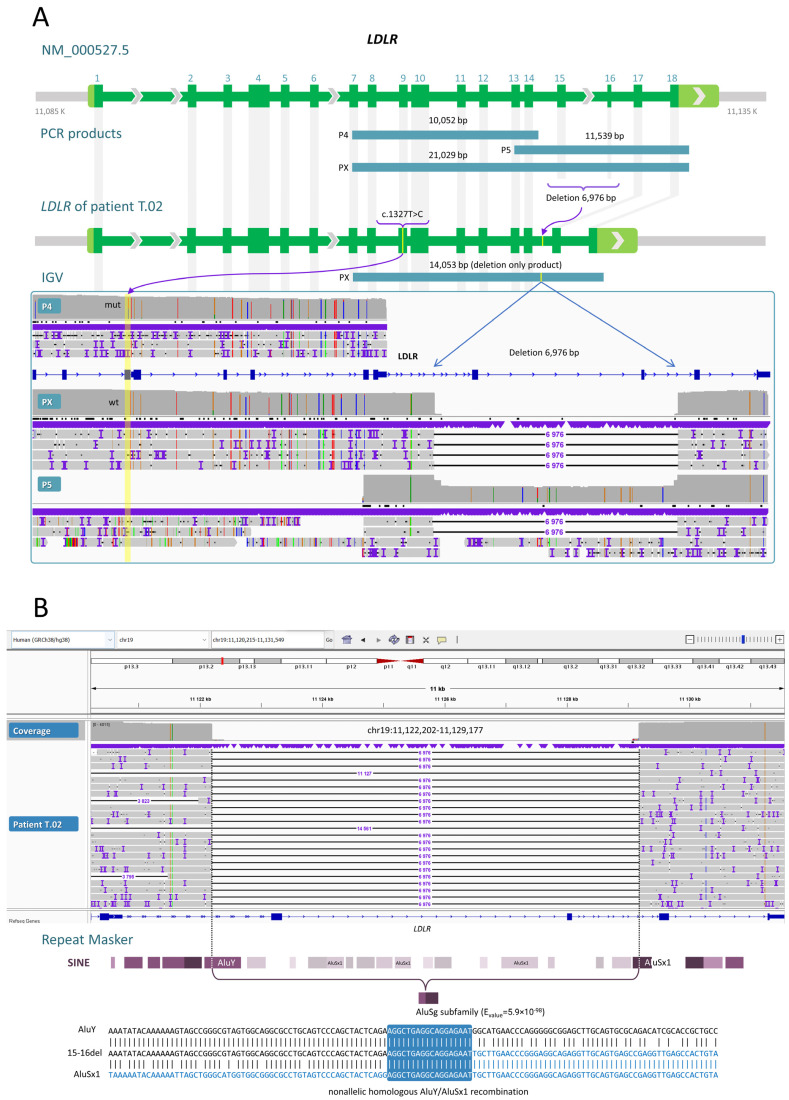
A snapshot from the IGV software showing the rare pathogenic variants of the *LDLR* gene in the T.02 sample. (**A**) ONT long reads identify a heterozygous state of the 6976 bp deletion that includes exons 15 and 16 (the amplicon of primer pair P5). Detection and phasing of missense variant c.1327T>C (wt/mut) relative to the 6976 bp deletion (amplicons of primer pairs P4, P5, and PX); (**B**) The precise location of breakpoints of the 6976 bp deletion and its DNA sequence are shown. The allele with the 6976 bp deletion has a higher probability of amplification with primer pair PX. Thus, only variant-containing reads are shown. *Alu* element homology is a possible cause of the deletion breakpoints.

The ability of our workflow to detect SVs can be illustrated using the results from sample T.02. We obtained two PCR products with primer pairs P5 and PX. A heterozygous 6976 bp deletion was found in sample T.02 between introns 14 and 16; it completely removed exons 15 and 16 (Figure 3A). The deletion removed amino acid residues 714 to 796 (without shifting the reading frame) located within the O-linked carbohydrate and membrane-spanning domains. This change to the protein is likely pathogenic.

Approximate breakpoints of this large deletion were determined by MPS. Through nanopore sequencing, we identified the precise location of the breakpoints: chr19:11,122,202-11,129,177 (GRCh38). Both deletion breakpoints are localized to repetitive elements *AluY* and *AluSx1* (Figure 3B). There is extensive sequence identity between the deletion breakpoints; this observation points to the mechanism of nonallelic homologous recombination (NAHR) between similar *Alu* elements.

No other pathogenic SVs were found in our patients.

### 2.3. Direct Reconstruction of the LDLR Haplotype

One of advantages of using ONT in this study is the phasing of all types of genetic variants. We found that all six pathogenic variants of three patients with compound heterozygous FH are in a *trans* configuration. For example, the *LDLR* gene fragment from exon 7 to exon 18 was PCR-amplified from genomic DNA (sample T.02) with primers (P4, P5, and PX). We detected missense mutation c.1327T>C (mut) in exon 9 and the 6976 bp deletion of exons 15 and 16 in different alleles (Figure 3A). These mutations are ~8.8 kb apart.

**Figure 4:**
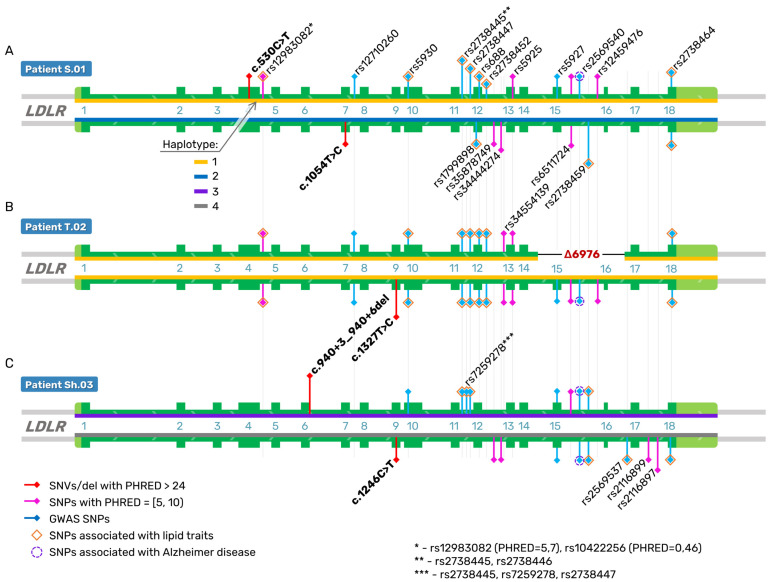
Detection and phasing of five rare pathogenic variants and common SNPs of *LDLR* gene with the highest CADD score statistic (PHRED) in the patients with compound heterozygous FH. (**A**) Haplotype structure of *LDLR* gene of S.01 patient. (**B**) Haplotype structure of *LDLR* gene of T.02 patient. (**C**) Haplotype structure of *LDLR* gene of Sh.03 patient.

The *trans*-heterozygous association between mutations c.1246C>T (exon 9) and c.940+3_940+6del (intron 6) of *LDLR* was also confirmed in patient Sh.03 (Figure 4; Table S1). Patient S.01 has one mutation, c.530C>T, in exon 4, and a second one (c.1054T>C) in exon 7 in a *trans* configuration (Figure 4; Table S1).

It should be noted that parents of two probands (T.02 and Sh.03) are presumed to be heterozygous for one pathogenic variant of the *LDLR* gene according to pedigree analysis. Unfortunately, biological samples from parents of all patients with compound heterozygous FH are not available.

At the next step of our analysis, we used the CADD tool to predict the deleteriousness of both exonic and intronic variants of the *LDLR* gene in the three patients with FH. All five rare pathogenic variants have high PHRED scores (greater than 24); these are missense mutations c.530C>T, c.1054T>C, c.1246C>T, and c.1327T>C, and one noncoding short deletion c.940+3_940+6del (Figure 4; Table S1).

In addition to analyzing rare mutations, we also examined haplotype structure of the *LDLR* gene in our three patients with FH by means of the common single-nucleotide polymorphisms (SNPs) that are associated with relevant traits according to GWASs. We also calculated the CADD score statistic for all of these patients’ genetic variants and visualized SNPs with the highest PHRED scores (Figure 4; Table S1). We noted extended haplotypes comprising 24 common SNPs across a 26.2 kb region (Figure 4; Table S1).

Most of these SNPs are noncoding, except for five synonymous variants rs5930 (p.Arg471=), rs1799898 (p.Leu575=), rs688 (p.Asn591=), rs5925 (p.Val653=), and rs5927 (p.Arg744=). Thirteen SNPs correlate with lipid traits (total cholesterol, LDL cholesterol [LDL-C], and ApoB levels) according to the GWAS catalog. SNPs rs2738447 and rs2738464 are also associated with CAD. Two common variants [rs5927 (p.Arg744=) and rs2569540] correlate with cortisol levels (saliva) and an Alzheimer’s disease polygenic risk score and with hepatitis C virus load, respectively.

Nine SNPs (rs12983082, rs35878749, rs34444274, rs34554139, rs5925, rs6511724, rs12459476, rs2116899, and rs2116897) have a PHRED score 5–10. Four SNPs (rs35878749, rs34444274, rs34554139, and rs6511724) are located in *Alu* elements (*AluSz6*, *AluSg*, and *AluSx3*). Eight SNPs are intronic variants, and rs5925 is a synonymous mutation. These SNPs are expression quantitative trait loci (eQTLs) for *LDLR* and *SMARCA4* in the blood according to the NESDA NTR Conditional eQTL Catalog [21].

Judging by HaploReg data, synonymous SNP rs5925 overlaps an RNA polymerase II–binding site in a liver cell line (HepG2); this location may indicate an enhancer site that could mediate altered *LDLR* expression [22].

Two SNPs, rs35878749 and rs34444274, are located in *AluSz6* elements within intron 12 having enhancer activity in the liver, fetal adrenal glands, and brain; these SNPs change the motif of transcription factors, including SREBP and HNF4, known to regulate transcription of *LDLR* in the liver. Two common variants—rs2116899 and rs2116897—are located in intron 17 of *LDLR* and are bound by proteins CTCF, ELF1, HEY1, HNF4A, HNF4G, P300, POL2, and RAD21 in the HepG2 cell line [22].

Mutations c.530C>T (p.Ser177Leu) and c.1327T>C (p.Trp443Arg) are on the haplotype that contains mainly alternative alleles. The other two rare coding variants are c.1054T>C (p.Cys352Arg) and the 6976 bp deletion of exons 15 and 16 and are affiliated with a different haplotype, which mainly contains reference alleles. The genotype of patient Sh.03 has fewer alternative alleles of common SNPs than patients S.01 and T.02. It should be noted that *LDLR* haplotypes having rare pathogenic variants contain both SNPs associated by GWASs with altered lipid levels and potentially functional SNPs that modulate *LDLR* expression or splicing.

## 3. Discussion

In recent years, a number of molecular diagnostic techniques for FH were created, including MPS, which is the most robust method for high-throughput sequencing of short DNA fragments [12]. There are several pipelines for targeted *LDLR* sequencing by MPS with relatively high sensitivity of SV detection owing to enrichment of the panel with the intronic content and optimization of bioinformatic algorithms [12,20].

Nevertheless, the main disadvantage of MPS is poor power for SV detection and the inability to phase genetic variants. The long-read sequencing method, on the contrary, can be applied to SV calling and direct haplotype reconstruction. To date, however, there has been only one study involving a practical application of long-read sequencing of a promotor and all coding regions of the *LDLR* gene by ONT [15]. However, the sequencing of introns 1, 6, 12, and 15 has not been performed in this work. Thus, it has not been possible to obtain information covering 20 kb of the *LDLR* gene in total. Before our work, there was also a problem with the phasing of genetic variants and direct haplotype analysis because of a lack of overlap among amplicons.

In our study, we designed five long-range PCRs to cover the *LDLR* gene from exon 2 to exon 18, including intronic sequences. We carefully designed the primers for long-range PCR because intron sequences of the *LDLR* gene are rich in *Alu* repeats [9,18]. Primer pairs were designed to detect exons 2–6, 4–11, 7–14, and 13–18. There was solid overlapping among four amplicons. Thus, we were able to detect the full spectrum of genetic variants in the *LDLR* gene from exon 2 to exon 18 with introns and to phase these variants in one run.

Then, long-range *LDLR* amplicons of three patients with compound heterozygous FH were sequenced using Oxford Nanopore MinION. As a result, all causative variants, including SNVs (c.530C>T, c.1054T>C, c.1246C>T, and c.1327T>C), small and large deletions (c.940+3_940+6del and the 6976 bp deletion of exons 15 and 16) and their correct zygosity were identified; these data showed high concordance with the results of MPS and Sanger sequencing. It was also possible to accurately determine breakpoints of the 6976 bp deletion. We found that the origin of this *LDLR* deletion is related to *Alu* elements, and that NAHR is responsible for this SV. NAHR has been described as a prevalent mechanism affecting SVs of the *LDLR* gene [11].

Judging by other reports, missense mutations c.530C>T, c.1054T>C, c.1246C>T, and c.1327T>C in *LDLR* can cause FH independently. For example, heterozygosity of the c.530C>T mutation in the *LDLR* gene is associated with FH in different countries, such as India [23], Portugal [24], Spain [25], Poland [26], and the Czech Republic [27]. Furthermore, this mutation in compound heterozygosity with EX7_EX10del (c.941-?_1186+?del) of the *LDLR* gene has been reported in Brazil [28] and in combination with p.Asp19His of the *ABCG8* gene in FH patients in Malaysia [29].

Pathogenic variant c.1054T>C has been found in heterozygous FH in Taiwan [30] and Russia [31], and in compound heterozygosity with p.Asp266Asn in a patient with FH in Western Siberia (Russia) [32]. Furthermore, heterozygosity of the c.1327T>C mutation of the *LDLR* gene correlates with FH in Russia [31,33]. There is evidence of mutation c.1246C>T in patients with heterozygous FH in Russia [31,32], Korea [34], and Taiwan [30]. Finally, large (6976 bp, exons 15–16) and small (4 bp, intron 6) deletions have been documented only in Russian patients [31,35].

In our study, we show that parents of patients T.02 and Sh.03 had FH. Thus, we can guess that mutations reside in different alleles (in a *trans* configuration). Long-read sequencing helped us phase all six genetic variants and confirmed their *trans* arrangement. To our knowledge, the exact *trans* positioning of these compound heterozygous mutations of the *LDLR* gene has not been reported elsewhere. The present study also confirms that these compound heterozygous mutations result in a severe clinical manifestation of FH.

For example, a 36-year-old female (patient S.01) with severe FH and CAD investigated in our study carries two missense mutations: c.530C>T (rs121908026) in exon 4 and c.1054T>C (rs879254769) in exon 7 (Table 1). The proband presented with myocardial infarction at 30 years of age in addition to tendon xanthomas, xanthelasma, lipoic corneal arcus, and high levels of total cholesterol and LDL-C.

Proband T.02 is a 31-year-old woman with xanthomas and severe coronary and carotid atherosclerosis with an extremely high concentration of total cholesterol and LDL-C before and even after treatment (23 and 17.6/15.2 mmol/L, Table 1). She was found to be compound heterozygous for a large deletion (c.2141-966_2390-330del, 6976 bp, exons 15 and 16) and a pathogenic missense variant (c.1327T>C, rs773566855) in exon 9 of the *LDLR* gene.

The third patient, Sh.03, is a 36-year-old woman with severe FH and CAD. It should be pointed out that she manifested a better response to lipid-lowering therapy than the other two patients (S.01 and T.02; Table 1). Patient Sh.03 carries two pathogenic variants—c.1246C>T (rs570942190) and c.940+3_940+6del (4 bp, intron 6)—of the *LDLR* gene in a *trans* configuration.

It is believed that *LDLR* mutations are concentrated in exon 4 because it is the largest exon in the gene, or because variants in this exon (encoding the ligand-binding domain) have a highly deleterious effect on gene function [36]. Patient S.01 carries pathogenic variant c.530C>T, which results in a substitution of serine by a leucine residue at position 177 (p.Ser177Leu) and affects the ligand-binding domain of LDLR. It has been demonstrated that this amino acid change has the most substantial impact on this protein’s function because of impaired LDL-C–binding activity and lowered LDL-C uptake; therefore, it is classified as a type 3 mutation [37,38].

In contrast, the mutation frequency in exons 15 and 16 is extremely low [36]. The effect of these mutations on FH pathophysiology has not been fully elucidated [39,40,41]. According to our study, patient T.02 has a deletion of *LDLR* exons 15 and 16 that eliminates amino acid residues 714 to 796, which are located within the O-linked carbohydrate and membrane-spanning domains of the protein. We can theorize that this deletion causes the retention of the mutant LDLR in the Golgi apparatus, underexpression of this protein on the plasma membrane, and a reduced ability of the LDLR protein to take up LDL-C.

Unfortunately, the lack of information on precise breakpoints of most SVs of the *LDLR* gene makes it impossible to establish whether the deletions we describe are identical to the ones reported from other populations. Nevertheless, deletions involving exon 15 (FH-Espoo) and exons 16 and 17 (FH-Helsinki) in the *LDLR* gene in a heterozygous state have also been seen in Russia and other populations, mainly in Northern Europe [32,42,43,44].

Mutations in the EGF precursor homology domain constitute 51.7% of all the missense variants described in *LDLR* [6]. It has been shown that these mutations are class 2 (partial or complete retention of LDLR in the endoplasmic reticulum), class 3 (defective binding to apolipoprotein B [apoB]), and class 5 (diminished LDLR recycling capacity). Our three patients carry missense mutations [c.1054T>C (p.Cys352Arg), c.1246C>T (p.Arg416Trp), and c.1327T>C (p.Trp443Arg)] in the EGF precursor homology domain.

Missense variant c.1246C>T (in exon 9) replaces arginine with tryptophan in codon 416 (p.Arg416Trp) in the β-propeller of the EGF precursor homology domain, and consequently LDLR fails to release LDL in the endosome, and thus the mutant receptor is not recycled to the cell surface; therefore, this variant is classified as a type 5 mutation [45]. Further functional studies are necessary to identify the mechanism of action of another two mutations—p.Cys352Arg and p.Trp443Arg—in this domain of the LDLR.

Patient Sh.03 has both missense variants c.1246C>T in exon 9 and a 4 bp deletion in intron 6 (c.940+3_940+6del) of the *LDLR* gene. According to SpliceAI, this variant has a score of 0.98 in terms of a donor loss and may influence splicing via skipping of exon 6 and the loss of extracellular LDLR class A repeat 7; these data confirm Semenova et al.’s in silico functional annotation [35]. Further biological research is needed to determine the mechanism underlying impairments of protein functions for these compound heterozygous mutations.

It has been shown that common SNPs in the *LDLR* gene have multiple effects on LDL receptor function. For example, the minor allele of synonymous SNP rs688, which is located in the β-propeller region of *LDLR*, correlates with increased alternative splicing of exon 12 and an altered gene transcript as well as impairment of LDLR endosomal recycling and/or PCSK9 binding [46,47]. Furthermore, there is evidence of mutual effects between rs688 and another synonymous SNP (rs5925) in the regulation of LDLR splicing efficiency, both in vitro and in vivo [48].

Noncoding SNPs in *LDLR* have also been reported to be functional; for example, rare and common variants located in the promoter region or intronic enhancer elements can abrogate or modify binding of nuclear transcription factors thereby leading to changes in *LDLR* expression [49,50]. On the other hand, the analysis of biological functional significance of such variants is complex because of a linkage disequilibrium (LD) between the SNPs that are coinherited with causal variants.

For the first time, we reconstructed ONT-based haplotypes of the *LDLR* gene of three patients with compound heterozygous FH on the basis of common SNPs associated mainly with LDL-C levels in GWASs and SNPs with the highest PHRED score (5–10) [4,51]. Finally, to test whether these SNPs affect gene expression levels, we searched for relevant data in NESDA NTR Conditional eQTL Catalog and HaploReg. In doing so, we found putative functional effects related to common SNPs rs5925 (exon 13), rs35878749, and rs34444274 (intron 12), rs2116899, and rs2116897 (intron 17). These SNPs have not been reported to be associated with lipid levels in a GWAS. Nonetheless, there is LD between these potentially functional SNPs and GWAS SNPs.

For example, a minor allele of variant rs688, an exon-splicing enhancer, has been reported to correlate with an increase in plasma total cholesterol and LDL-C levels in several independent populations [4]. High LD between rs688 and rs5925 among Europeans has been documented by Gao F. et al. and Caruz A. [46,52]. In the present study, we detected an *LDLR* haplotype that contains minor alleles of both synonymous SNPs rs688 and rs5925 but reference alleles of rs35878749 and rs34444274 (Figure 4; Table S1).

*LDLR* gene expression is controlled mainly by *cis*-regulatory elements in the 3′ untranslated region (UTR) via changes in mRNA stability [53]. Variant rs2738464 is present in the 2.5-kb 3′UTR of the *LDLR* gene and correlates with total cholesterol and LDL-C levels as well as risks of CAD and myocardial infarction [4]. In our study, two SNPs—rs2116899 and rs2116897—located in intron 17 affect the binding of various transcription factors in the HepG2 cell line and alter *LDLR* expression in the blood [21,22].

Recently, it was found that there are large effects of rare *LDLR* variants in introns 2, 3, 16, and 17, namely, markedly elevated LDL-C levels in ancestrally diverse individuals; these effects are similar to those of rare coding mutations [54]. Rare noncoding variants have been identified in intron 14 in patients with FH [10,55]. In our paper, we identified common SNPs in introns 12, 15, and 17, which can be functionally significant in the regulation of *LDLR* expression and alternative splicing.

Intronic *Alu* elements may contribute to alternative splicing and natural mRNA isoform diversity and can alter splicing efficiency and transcript levels in disease phenotypes [56,57]. Notably, we found that intronic SNPs rs35878749, rs34444274, rs34554139, and rs6511724, which are located in *Alu* elements (*AluSz6*, *AluSg*, and *AluSx3*), have the highest PHRED scores among other common SNPs. On the basis of in silico prediction tools (NESDA NTR Conditional eQTL Catalog and HaploReg), we can hypothesize that these *Alu*-associated genetic variants can have regulatory potential and are interesting research directions to pursue.

There are several limitations of the present study that must be considered. Due to the large size and *Alu* complexity of the analyzed genomic locus, we could not amplify the region encompassing intron 1 of the *LDLR* gene, where *cis*-acting gene regulatory sites are commonly found. The mechanism of detrimental effects of six pathogenic variants and common potentially functional SNPs of the *LDLR* gene were analyzed here only using literature sources and bioinformatic tools. Unfortunately, family-based cascade genetic screening of FH could be performed only for patient S.01. Her mother and daughter with FH carry the p.Ser177Leu mutation in exon 4 of the *LDLR* gene. Further research into the specific function of these genetic variants, both individually and in a phasing state, would be of great value in determining the extent to which they regulate lipid levels.

Lastly, analyses involving a larger number of healthy individuals and patients with Mendelian FH, common lipid-metabolism-related disorders such as CAD, and Alzheimer’s disease can give us a greater insight into variations of the *LDLR* gene at the population level in different ethnic groups and will be helpful for early prevention or prognosis of these disorders. We think that directly extended haplotype reconstruction of the *LDLR-SMARCA4* locus of patients with FH may explain its negative association with CAD [58]. Because LDLR contributes to both cholesterol and amyloid-β homeostasis, insights into the variation of *LDLR* and splicing regulation in different cell types of target organs may clarify the co-occurrence of cardiovascular diseases and Alzheimer’s disease.

## 4. Materials and Methods

### 4.1. Patient Characteristics

Three adult female patients (age range 31–36 years) with genetically confirmed FH, who were regularly followed at a specialized FH center of the Federal State Budgetary Institution National Medical Research Center of Cardiology Named after Academician E.I. Chazov (Ministry of Health of the Russian Federation; Moscow), were recruited into the study during their annual medical examinations. The FH patients had previously gotten this diagnosis in accordance with accepted standard criteria as described in ref. [59]. Clinical signs of FH in these patients are presented in Table 1. For the current study, all clinical and laboratory data were collected from the patient’s medical histories.

*LDLR* mutations were found by MPS using a custom capture library (63 dyslipidemia genes) and Illumina HiSeq 1500 [31,35,60,61]. All genetic variants, except the large deletion (c.2141-966_2390-330del, 6976 bp, introns 14–16), were confirmed by Sanger sequencing as described before [62].

### 4.2. DNA Extraction and Long-Range PCR

Genomic DNA of patients was isolated from peripheral-blood samples using the Monarch^®^ HMW DNA Extraction Kit for Cells & Blood (New England BioLabs, Ipswich, MA, USA), followed by assessment of the concentration and purity of the isolated DNA on NanoDrop 8000 (Thermo Fisher Scientific, Waltham, MA, USA) and by electrophoresis in a 0.8% agarose gel.

The *LDLR* primers for the long-range PCR were designed by means of PrimerQuest [63] and checked in the OligoAnalyzer Tool (https://eu.idtdna.com/pages/tools/oligoanalyzer, accessed on 28 April 2022) [64].

The long-range PCR for amplifying each *LDLR* gene fragment was conducted in a 25 μL reaction mixture containing 12.5 μL of LongAmp Taq 2X Master Mix (New England BioLabs), 5.0 μL of 5X SE PCR Stabilizer (SibEnzyme, Novosibirsk, Russia), 0.5 μM (final concentration of) each primer in a pair (Table 2), and 50 ng of genomic DNA.

The long-range-PCR program was as follows: initial denaturation at 94 °C for 4 min; 35 cycles of denaturation at 94 °C for 20 s, primer annealing at 60 °C for 20 s, and elongation at 68 °C for 12 min (after 10 cycles, adding an increment of +10 s/cycle to the elongation step), followed by final elongation for 10 min at 68 °C. The PCR products were visualized by 1% agarose gel electrophoresis.

### 4.3. Library Preparation and ONT Sequencing

Concentrations of the amplified gene fragments were evaluated using the BR dsDNA Qubit Kit (Thermo Fisher Scientific). For each patient, all PCR products (100 fmol each) were pooled at equimolar concentrations (48 µL final volume) and used for library preparation using the Native Barcoding Amplicons Kit (EXP-NBD104, EXP-NBD114, and SQK-LSK109; Oxford Nanopore Technologies, Oxford, United Kingdom) according to the manufacturer’s protocol. The prepared library was loaded into a MinION flow cell (FLO-MIN106D; Oxford Nanopore Technologies), and the sequencing was carried out for 48 h.

### 4.4. Bioinformatic Analyses of Nanopore Sequencing Data

Base calling and demultiplexing of the data were performed in the Guppy v.5.0.7 software [65]. Reads of the amplicons of the *LDLR* gene were aligned to the human genome build GRCh38.p13 using MiniMap2 [66]. Generated SAM files were converted to BAM format in SAMtools [67]. The minimum sequencing depth was found to never dip below 150× according to Bedtools “coverage” [68]. The variant-calling and phasing steps were performed by algorithms Clair3 and Sniffles2 [69]. Data were viewed in IGV v.2.15.2 [70]. MultiQC v.1.12 was used to generate data sequencing statistics and quality metrics [71].

### 4.5. Sanger Sequencing of the Promoter and Exon 1 of the LDLR Gene

For the promoter and exon 1 of the *LDLR* gene, we carried out classic PCR to enrich this part of the gene with primers P1 F: 5′-CGGAGACCCAAATACAACAAATC-3′ and R: 5′-TTTCCCTTAAATCCCTCAGACTC-3′. The amplicon size was 587 bp. The DNA samples were sequenced with the BigDye Terminator v3.1 Cycle Sequencing Kit on an Applied Biosystems 3730 Genetic Analyzer (Thermo Fisher Scientific). The results were interpreted with the help of Chromas 2.6.3 software (Technelysium, South Brisbane, QLD, Australia).

### 4.6. In Silico Assessment of Pathogenicity and Regulatory Potential of the Variants

The identified genetic variants were evaluated in terms of their effect on protein structure and/or function using web-based annotation tools and databases (Annovar, PolyPhen2, SIFT, Mutation Tester, MutPred, gnomAD, RUSeq, dbSNP, and HGMD). Pathogenic variants were also manually subjected to searches in PubMed and VarSome [72]. The pathogenicity of the genetic variants was assessed based on guidelines for the interpretation of high-throughput sequencing data [73,74].

In addition, potential splice effects of intronic variants were assessed in SpliceAI [75]. The Δ Score was obtained with default parameters. CADD v.1.6 was utilized for predicting the deleteriousness of both exonic and intronic variants [51]. Common SNPs were run through HaploReg v4.1 and NESDA NTR Conditional eQTL Catalog to assess their functional consequences [21,22].

To identify rare pathogenic genetic variants and common potentially functional SNPs, we chose variants with the highest CADD score statistic (PHRED) and integrated them with the sequence context (*Alu* elements), transcription factors and histone marks (HaploReg v4.1), and blood eQTLs (NESDA NTR Conditional eQTL Catalog).

## 5. Conclusions

To the best of our knowledge, this is the first ONT study on FH to cover the *LDLR* gene from exon 2 to exon 18 with introns, and it should make it efficient to determine a nearly complete analysis of this gene. Therefore, we were able to detect both coding and noncoding variants, such as SNVs and small and large deletions. In introns 12, 15, and 17, we also identified common SNPs that can be functionally significant in the regulation of *LDLR* expression and alternative splicing. The long reads allowed for the phasing of the genetic variants and for direct haplotype analysis of the *LDLR* gene at an individual level without knowledge about their inheritance from parents.

The ability to detect the full spectrum of genetic variants in *LDLR* is critical not only for making a molecular diagnosis of FH but also for research. This is because the variation and extended haplotype structure of *LDLR* in different ethnic groups remains largely unknown, for example, in patients with altered lipid metabolism, Mendelian FH, and common diseases (CAD and Alzheimer’s disease).

## Figures and Tables

**Table 1:** Characteristics of the patients with FH.

Subjects’ IDs:	S.01	T.02	Sh.03
Sex	Female	Female	Female
Age, years	36	31	36
Family history of FH or CAD	Yes (mother and daughter with FH; brother, MI under 50 y.o.)	Yes (father and mother both with FH; maternal uncle with FH)	Yes (father and mother both with FH)
Total cholesterol, mmol/L (before/after treatment)	19/15.64–17.61	23/16.73–18.41	17.25/5.31–4.84
LDL-C, mmol/L (before/after treatment)	17/14.45–16.24	NA/15.2–17.25	15.15/3.86–2.98
Triglycerides, mmol/L (before/after treatment)	NA/0.62–0.86	NA/1.67–1.1	NA/0.57–0.75
Tendon xanthomas	Yes	Yes	Yes
Xanthelasma	Yes	Yes	No
Lipoic corneal arcus	Yes	No	No
Clinical and instrumental manifestations of CAD	Yes (MI at 30 y.o.)	Yes (CABG at 23 y.o., CAS at 26 y.o., TAVI at 31 y.o., death from COVID-19 at 32 y.o.)	Yes (CABG at 26 y.o.)
Lipid-lowering therapy	Rosuvastatin 40 mg, Ezetimibe 10 mg. Inclisiran 300 mg.	Rosuvastatin 40 mg, Ezetimibe 10 mg. Inclisiran 300 mg.	Rosuvastatin 40 mg, Ezetimibe 10 mg. Inclisiran 300 mg.
Mutations in the *LDLR* gene (NM_000527.5)	c.530C>T or FH Puerto Rico (exon 4)/c.1054T>C (exon 7)	c.2141-966_2390-330del (6976 bp deletion of exons 15–16)/c.1327T>C (exon 9)	c.1246C>T (exon 9)/c.940+3_940+6del (4 bp, intron 6)
Variant annotation in silico (Varsome)	Pathogenic/Pathogenic	-/Pathogenic	Pathogenic/Likely Pathogenic
Amino acid change (NP_000518.1)	p.Ser177Leu/p.Cys352Arg	p.Glu714_Ile796del/p.Trp443Arg	p.Arg416Trp/NA
rsID	rs121908026/rs879254769	NA/rs773566855	rs570942190/NA

FH: familial hypercholesterolemia, CAD: coronary artery disease, MI: myocardial infarction, CABG: coronary artery bypass grafting, CAS: carotid artery stenting, TAVI: transcatheter aortic valve implantation, NA: not applicable or not available.

**Table 2:** The primer pairs targeting parts of *LDLR*.

ID	PCR Products	Forward (5′-3′)	Reverse (5′-3′)	Expected Length (bp)
P2	Intron 1 to intron 6	GTTCCTTCTTTGTGTCCTCCA	GTCTTTCAGTATCCACCACAGAG	8618
P3	Intron 3 to intron 11	TGGTGTTGGGAGACTTCACA	CTCTCCAATGGGCAGGTAGG	11,520
P4	Exon 7 to intron 14	CCTTAAGATCGGCTACGAGTG	TCAGAAATCAGATCACCTCTTCAG	10,052
P5	Exon 13 to 3′UTR	GAGGATATGGTTCTCTTCCACAA	GGCTTAGAGATTGGTGGATGAG	11,539
PX	Exon 7 to 3′UTR ^1^	CCTTAAGATCGGCTACGAGTG	GGCTTAGAGATTGGTGGATGAG	21,029

^1^ A combination of P4 forward and P5 reverse. The purpose of this primer pair is to detect large deletions between exons 7 and 18.

## Data Availability

Raw data are available from the corresponding author upon request.

## Supplementary Materials

The supporting information can be downloaded at: https://www.mdpi.com/article/10.3390/ijms24054471/s1.
